# Presymptomatic studies in genetic frontotemporal dementia

**DOI:** 10.1016/j.neurol.2013.07.010

**Published:** 2013-10

**Authors:** J.D. Rohrer, J.D. Warren, N.C. Fox, M.N. Rossor

**Affiliations:** Dementia Research Centre, UCL Institute of Neurology, University College London, Queen Square, London, WC1N 3BG, UK

**Keywords:** Frontotemporal dementia, Primary progressive aphasia, Progranulin, Tau, C9orf72, Dégénérescence lobaire frontotemporale, Démence frontotemporale, Aphasie progressive primaire, Progranuline, MAPT, C9ORF72

## Abstract

Approximately 20% of patients with the neurodegenerative disorder frontotemporal dementia (FTD) have an autosomal dominant pattern of inheritance. Genetic FTD is caused by mutations in three genes in most cases (progranulin, microtubule-associated protein tau and chromosome 9 open reading frame 72) although a number of other genes are rare causes. Studies of other neurodegenerative diseases have shown imaging and biomarker evidence of disease onset many years prior to the development of symptoms. Similar studies in genetic FTD are now revealing evidence of a series of presymptomatic changes, initially in plasma biomarkers followed by MR imaging abnormalities of functional and structural connectivity and then grey matter atrophy. Lastly, neuropsychometric tests become abnormal in proximity to the onset of symptoms. Such studies have been relatively small until now but research centres with an expertise in genetic FTD are now forming consortia such as the Genetic Frontotemporal Dementia Initiative (GenFI) to create larger cohorts that can form the basis of future clinical trials.

## Résumé

Les démences frontotemporales (DFT), dans environ 20 % des cas, résultent d’un mode de transmission autosomique dominant. Les trois causes génétiques les plus fréquentes sont des mutations des gènes *GRN, MAPT* et des expansions hexanucléotidiques au sein du gène *C9orf72*, alors que d’autres gènes sont plus rarement impliqués. Des études dans d’autres maladies neurodégénératives ont montré que des marqueurs biologiques et d’imagerie du début de la maladie précèdent de plusieurs années les premiers symptômes. Des études similaires concernant les formes génétiques des DFT mettent actuellement en évidence des modifications présymptomatiques, initialement des biomarqueurs plasmatiques, suivies des modifications en imagerie de la connectivité fonctionnelle et structurelle puis des signes d’atrophie de la substance grise. Enfin, les performances aux tests neuropsychologiques deviennent anormales dans la période proche du début des symptômes. Ces études restent peu nombreuses mais les centres ayant une expertise dans la génétique des DFT se regroupent actuellement en consortium tel que l’Initiative pour la Génétique des Démences FrontoTemporales (GenFI) afin de disposer de larges cohortes qui serviront de base au développement des futurs essais cliniques.

Frontotemporal dementia (FTD) is a neurodegenerative disorder usually presenting with either behavioural or language impairment, although it has significant overlap with motor neurone disease (MND) and the atypical parkinsonian disorders (Seelaar et al., 2011). Around 20% of patients within the FTD spectrum have an autosomal dominant pattern of inheritance with mutations in the genes progranulin (*GRN*), microtubule-associated protein tau (*MAPT*) and chromosome 9 open reading frame (*C9orf72*) being the commonest causes (Rohrer et al., 2009, Rohrer and Warren, 2011). These each account for about 5–10% of all FTD cases although there is geographical variability, e.g. in a US series, *C9orf72* expansions were the most common cause (7% of all FTD, compared to 5% *GRN* and 4% *MAPT*) (Dejesus-Hernandez et al., 2011) whereas in a Dutch series, *MAPT* mutations were the most common cause (10% of all FTD, compared to 9% *C9orf72* and 7% *GRN*) (Simón-Sánchez et al., 2012), and in a UK series, the frequency of mutations in each of the genes was approximately equal (7% *C9orf72*, 7% *GRN*, 6% *MAPT*) (Mahoney et al., 2012). Mutations in other genes (*VCP*, *CHMP2B*, *FUS*, *TARBP*, *DCTN1* and *SQSTM1*) have been described as causing a disorder within the FTD spectrum but occur with a very low frequency (Rohrer and Warren, 2011, Rubino et al., 2012).

*GRN*, *MAPT* and *C9orf72* mutations can all cause behavioural variant FTD and this is the most common presenting syndrome for each of the genes (Rohrer and Warren, 2011, Dejesus-Hernandez et al., 2011, Renton et al., 2011). However, there are other clinical syndromes that are seen more frequently in association with one gene rather than another. MND is seen commonly in association with expansions in *C9orf72* (although can rarely occur with *GRN* mutations) whilst primary language impairment (usually a progressive nonfluent aphasia, PNFA) is seen with *GRN* mutations (and rarely with expansions in *C9orf72*). Parkinsonism may be seen with any of the mutations with a variable phenotype, sometimes very similar to idiopathic Parkinson's disease (in *MAPT* mutations), in other cases, a corticobasal syndrome (with *GRN* or, less commonly, *MAPT* mutations), and more rarely a progressive supranuclear palsy syndrome (with *MAPT* mutations) (Rohrer and Warren, 2011).

From a neuroimaging perspective, patients with *GRN* mutations tend to have an asymmetrical pattern of atrophy compared to the more symmetrical pattern seen in *MAPT* and *C9orf72* mutations (Rohrer et al., 2010, Whitwell et al., 2012). Early areas of involvement are the temporal, inferior frontal and inferior parietal lobes in *GRN* mutations and the anteromedial temporal and orbitofrontal lobes in *MAPT* mutations (Rohrer et al., 2010, Whitwell et al., 2012). *C9orf72* expansions have a more variable pattern of cortical atrophy (although usually frontotemporal predominant) with some studies suggesting that subcortical (particularly thalamic) and cerebellar atrophy are also seen (Mahoney et al., 2012, Rohrer and Rosen, 2013).

More detailed reviews of the clinical and neuroimaging features of symptomatic patients with genetic FTD are described elsewhere (Rohrer and Warren, 2011, Van Swieten and Heutink, 2008, Van Swieten and Spillantini, 2007). This paper reviews studies of those siblings and children of patients with genetic FTD who are currently clinically well but are at a 50% risk of developing a disorder within the FTD spectrum, often termed presymptomatic or ‘at-risk’ genetic FTD.

## 1 Why study presymptomatic genetic frontotemporal dementia?

Whilst there are currently no treatments that can delay the onset or prevent the progression of genetic FTD, there are promising avenues for treatment, particularly for *GRN* mutations where a uniform disease mechanism of loss of progranulin function operates in all mutation carriers. Drugs including chloroquine, nimodipine and vorinostat have been shown to increase progranulin concentration (Capell et al., 2011, Cenik et al., 2011) suggesting their use in future clinical trials. Ideally, therapies would be instituted when the minimum of irreversible neuronal loss has occurred. This makes trial design challenging and increases the importance of biomarkers in selecting suitable subjects and in monitoring progression, However, despite having potential disease-modifying therapies there are still no biomarkers of genetic FTD that can confidently predict when disease-modifying therapy should be initiated or how the response to it should be monitored. Evidence from other neurodegenerative diseases such as familial Alzheimer's disease shows that there are changes in a number of biomarkers many years before symptom onset (Bateman et al., 2012) suggesting that the ideal time for treating these disorders is likely to be prior to clinical presentation. The identification of robust biomarkers in genetic FTD that are indicative of disease onset and progression are therefore prerequisites for any disease-modifying therapy trial.

## 2 What do we know about presymptomatic genetic frontotemporal dementia currently?

A number of studies have examined presymptomatic genetic FTD although the majority of these have been either individual case reports or relatively small case series. Whilst a few have shown presymptomatic changes in neuropsychometric testing of executive function and social cognition (Janssen et al., 2005, Rohrer et al., 2008), most have not shown any cognitive changes.

Prior to manifesting neuropsychometric abnormalities and several years before disease onset, a number of structural T1 MR imaging studies have now shown evidence of grey matter atrophy. One study examined a patient with familial FTLD-U (now known to be associated with a *GRN* mutation) and showed evidence of very focal left frontal lobe atrophy affecting the pars opercularis about two years prior to the onset of PNFA (Janssen et al., 2005). Another single case report of a subject with a GRN mutation who later developed PNFA showed evidence of early left hemisphere atrophy, particularly in the frontal lobe but also in the middle and inferior temporal gyri and angular gyrus, at least eighteen months prior to symptom onset (Rohrer et al., 2008). This was consistent with a study of four presymptomatic *GRN* mutation carriers from a PNFA family who had a similar pattern of atrophy and also hypometabolism on FDG-PET scanning (Cruchaga et al., 2009). *MAPT* mutations have been studied less frequently, although one study did show presymptomatic hippocampal atrophy (Miyoshi et al., 2010).

Studies using other imaging modalities have identified presymptomatic changes that appear to occur earlier than grey matter atrophy. A small study of at-risk *GRN* mutation carriers (*n* = 4) showed no evidence of grey matter atrophy but using diffusion tensor imaging (DTI) did show reduced fractional anisotropy (FA) in the left uncinate fasciculus and inferior fronto-occipital fasciculus compared with controls (Borroni et al., 2008). A larger DTI study of presymptomatic *GRN* mutation carriers (*n* = 27) also showed decreased FA in the inferior fronto-occipital fasciculus (on the right) as well as involvement of the right anterior and superior corona radiata, anterior thalamic radiation, superior longitudinal fasciculus, forceps minor and internal capsule (Dopper et al., 2013). In comparison, presymptomatic *MAPT* mutation carriers (*n* = 9) showed widespread decreased FA (and also increased mean, axial and radial diffusivity) throughout frontotemporal white matter tracts.

This same study also examined changes in resting state functional MRI (RS-fMRI) in presymptomatic *GRN* and *MAPT* mutation carriers. Studies of RS-fMRI have been used to elucidate a series of coherent large-scale brain networks, the best described being the ‘default mode network’, a set of regions centred around the posterior cingulate, precuneus, lateral parietal and temporal lobes, and medial prefrontal cortex that routinely decrease their activity during attention-demanding tasks. In FTD, the network that has been studied in the most detail is a ‘salience network’ centred around the fronto-insula cortex and dorsal anterior cingulate. In the study by Dopper et al., no changes were seen in *MAPT* mutation carriers but there was reduced connectivity in the anterior midcingulate cortex (an area within the salience network) in *GRN* mutation carriers without any changes in the posterior cingulate cortex (an area within the default mode network) (Dopper et al., 2013). This is in contrast to another study of presymptomatic *GRN* mutation carriers which showed increased connectivity in a small area in the medial prefrontal cortex (which the authors attribute to the salience network) also without any changes in other networks (Borroni et al., 2012). Another study of presymptomatic *MAPT* mutation carriers showed reduced connectivity in parts of the default mode network (lateral temporal and medial prefrontal cortex) with increased connectivity in other parts of the default mode network (medial parietal) and no changes in the salience network (Whitwell et al., 2011). It remains unclear why there are such divergent findings in RS-fMRI studies although this may represent differences in the stage at which carriers were studied or in the methods used for analysis.

There has been a single study of magnetic resonance spectroscopy in *MAPT* mutation carriers showing metabolite abnormalities several years before the onset of symptoms (elevated myoinositol/creatine ratio and decreased N-acetylaspartate/myoinositol ratio) (Kantarci et al., 2010).

There are few established serum or CSF markers of FTD. Decreased plasma progranulin concentrations are found in symptomatic patients with *GRN* mutations but similarly low levels have also been found in presymptomatic mutation carriers who are in their 20's and 30's and are therefore many years prior to disease onset (Borroni et al., 2012, Ghidoni et al., 2012). It remains unclear whether other serum or CSF markers will be abnormal this early as has been found for a number of biomarkers in familial Alzheimer's disease (Bateman et al., 2012).

In summary, these studies suggest that there is a sequence of changes seen in different biomarkers of genetic FTD prior to clinical onset of symptoms: the earliest of these are likely to be a number of plasma (and CSF) markers (although apart from plasma progranulin concentration this remains unproven) followed by markers of functional and structural connectivity, then grey matter atrophy, and finally mild neuropsychometric abnormalities in proximity to the first symptoms.

## 3 How can we take things forward?

Genetic FTD is a rare condition and individual research centres have only been able to study relatively small numbers of participants. Further progress is therefore likely to depend on pooling populations of presymptomatic FTD, which allows both for a large cohort of individuals and for a range of different mutations within the same gene to be investigated. Larger studies also permit a greater understanding of the natural history of the disorder, from younger at risk subjects many years before symptom onset through to those subjects near to the time of conversion to manifest disease (thus allowing identification of ‘proximity markers’ to symptom onset). Head-to-head comparison of candidate biomarkers can be performed investigating whether combined multimodal indices may be more useful than individual markers, or whether novel indices should be used e.g. social cognition tasks that highlight cognitive changes prior to standard neuropsychometry, or fMRI studies that identify vulnerable brain networks. With a growing understanding of many neurodegenerative disorders as nexopathies targeting a particular network, large studies of genetic FTD will also provide unique insights into the underlying pathophysiology of the disease (Warren et al., 2012).

Successful models for multicentre consortia of this kind have been established for other neurodegenerative diseases e.g. the Dominantly Inherited Alzheimer's Disease Network (DIAN) and the TrackHD consortium for Huntington's disease (Bateman et al., 2012, Tabrizi et al., 2009). Based upon these models the Genetic Frontotemporal dementia Initiative (GENFI) was set up in 2011 to bring together research centres across Europe and Canada with an interest in clinical studies of presymptomatic genetic FTD. There are currently twelve centres that form the consortium, situated in the UK, Italy, Holland, Sweden and Canada, with over two hundred subjects now recruited. Subjects undergo a standardized clinical and neuropsychological assessment (including the revised Cambridge Behavioural Inventory (CBI-R) (Wear et al., 2008), Frontotemporal dementia Rating Scale (FRS) (Mioshi et al., 2010) and the Uniform Data Set (UDS) psychometric battery (Morris et al., 2006)) as well as volumetric T1, DTI, RS-fMRI and arterial spin labeling perfusion MRI. DNA, RNA, serum and plasma samples are taken from all subjects and some subjects also have cerebrospinal fluid collected. A secure online database is in place for uploading all data in a pseudoanonymised form. A particular ethical consideration is the recruitment of at risk subjects without the need for them to have undergone presymptomatic genetic testing. Protocols are in place to ensure that testing in those currently unaware of their genetic status is performed without the knowledge of the result becoming known either to them or to any clinician involved in assessing them (unless the subject wishes to know the result and has gone through a standard clinical process of genetic counseling). The aim of the study will be to create a large cohort of presymptomatic at risk genetic FTD subjects who could take part in clinical trials and who are studied using a uniform methodological platform and infrastructure, with the key outcomes being to develop robust markers of disease onset and stage including those indicative of optimal time to start disease-modifying therapy as well as markers of disease progression that can be used as outcome measures in clinical trials.

We are at a turning point in genetic FTD research where clinical trials of disease-modifying therapy are now a reality and it is therefore of the utmost important to have the right infrastructure in place and the correct biomarkers measured to ensure the best possible chance that such trials will succeed.

## Disclosure of interest

The authors declare that they have no conflicts of interest concerning this article.
